# Heterogeneity and Polygenicity in Psychiatric Disorders: A Genome-Wide Perspective

**DOI:** 10.1177/2470547020924844

**Published:** 2020-05-18

**Authors:** Frank R. Wendt, Gita A. Pathak, Daniel S. Tylee, Aranyak Goswami, Renato Polimanti

**Affiliations:** Department of Psychiatry, Yale School of Medicine and VA CT Healthcare Center, West Haven, CT, USA

**Keywords:** psychiatric disorders, heterogeneity, polygenicity, genome-wide association studies

## Abstract

Genome-wide association studies (GWAS) have been performed for many psychiatric disorders and revealed a complex polygenic architecture linking mental and physical health phenotypes. Psychiatric diagnoses are often heterogeneous, and several layers of trait heterogeneity may contribute to detection of genetic risks per disorder or across multiple disorders. In this review, we discuss these heterogeneities and their consequences on the discovery of risk loci using large-scale genetic data. We primarily highlight the ways in which sex and diagnostic complexity contribute to risk locus discovery in schizophrenia, bipolar disorder, attention deficit hyperactivity disorder, autism spectrum disorder, posttraumatic stress disorder, major depressive disorder, obsessive-compulsive disorder, Tourette’s syndrome and chronic tic disorder, anxiety disorders, suicidality, feeding and eating disorders, and substance use disorders. Genetic data also have facilitated discovery of clinically relevant subphenotypes also described here. Collectively, GWAS of psychiatric disorders revealed that the understanding of heterogeneity, polygenicity, and pleiotropy is critical to translate genetic findings into treatment strategies.

## Introduction

Genome-wide association studies (GWAS) are powerful tools for risk allele and gene discovery when applied to complex traits.^1^ The resulting data enable investigation of biological mechanisms, pathways, tissues, and cell types relevant for phenotype etiology,^2–4^ evolutionary pressures shaping genetic risk for a trait in the general population,^5–9^ and correlation and causal inference between traits.^10–15^

In psychiatry, GWAS have uncovered a high degree of polygenicity underlying mental illnesses and related complex phenotypes.^8,16–35^ Polygenicity describes the contribution of many single nucleotide polymorphisms (SNPs) with relatively small effect sizes to phenotype development. This phenomenon is ubiquitously observed in psychiatric disorders and comorbid phenotypes, as evidenced by the detection of tens to hundreds of genome-wide significant (GWS) linkage disequilibrium (LD) independent loci.^1^ Additionally, omnigenic models of complex traits suggest that highly interconnected gene regulatory networks influence trait etiology through a set of core genes and their associated regulatory elements and members of similar protein pathways.^36^

Though rare and structural variation contribute to the genetic liability of psychiatric disorders,^37–39^ the magnitude and ubiquity of GWAS data limit the scope of this review to common genetic variation. Here, we discuss GWAS-based methods for interrogating the etiology of psychiatric disorders. Next, we describe the polygenic nature of psychiatric disorders and how genetic and phenotype heterogeneity may affect our ability to detect risk loci for these traits. Finally, we briefly discuss future directions for the field of psychiatric genomics, including the advantage of large GWAS consortia and biobanks for exploring phenotype heterogeneity using genetic data.

## GWAS for Detecting the Polygenic Architecture of Psychiatric Disorders

The primary goal of human genetics is to identify risk and protective factors for disease. Many aspects of human health and disease pose a challenge toward this goal. Complex traits lack a single gene with large enough effects to study in singularity with generalizable findings. Conversely, GWAS of complex traits have revealed large degrees of polygenicity underlying mental health (i.e. psychiatric disorders, behavior, personality traits, social science traits, and brain region measurements).^1^ GWAS are hypothesis-generating experiments that detect relationships between allele frequency and categorical or quantitative phenotypes.^40,41^ The results of GWAS are typically displayed as a Manhattan plot with base-pair positions ordered per chromosome on the *x*-axis and significance (–log_10_(association *p* value)) on the *y*-axis, creating a densely populated plot mimicking the Manhattan skyline.

A successful GWAS requires a well-considered phenotype. Three diagnostic classification systems for psychiatric disorders are used worldwide.^42^ The Diagnostic and Statistical Manual of Mental Disorders (DSM, currently the 5th edition) was developed by the American Psychiatric Association and is used primarily to guide clinical practice and mental health research. The International Classification of Diseases (ICD) was developed by the World Health Organization (WHO) and is used by clinicians for charting patient diagnosis of both physical and mental health conditions.^43–45^ The application of each system varies widely throughout the world, where DSM is widely used in the United States in contrast to the predominance of ICD in Europe.^42^ While there is a high degree of overlap between these systems, there is clinical heterogeneity, for example, in classifying some psychiatric outcomes.^46,47^ Phenotype heterogeneity may alter the sample allele distribution leading to (i) false negatives (i.e. true differences in allele frequency are masked) or (ii) false positives (i.e. allele frequency differences due to unbalanced phenotype distribution).^48^ The Research Domain Criteria (RDoC) paradigm compliments DSM and ICD classification systems by assessing clinical phenotypes hypothesized to more closely map onto underlying biological systems (e.g. neuroimaging data^49^ and brain circuit activity^50^). RDoC-based approaches offer an alternative to heterogeneous diagnostic systems, by permitting assessment of negative and positive valence, cognitive systems, sensorimotor systems, social processing systems, and/or arousal and regulatory systems across persons affected by different disorders, as well as healthy comparison subjects. Psychiatric disorders are often diagnosed based on a heterogeneous combination of symptom counts (i.e. an individual endorses a subset of symptoms but may not meet all criteria) as well as meeting full diagnostic criteria. These features also may be assessed for lifetime prevalence or, for example, last month prevalence. Note that herein we summarize findings from large studies of psychiatric disorders assessed with different instruments and considering different diagnostic criteria.

**Figure 1.**
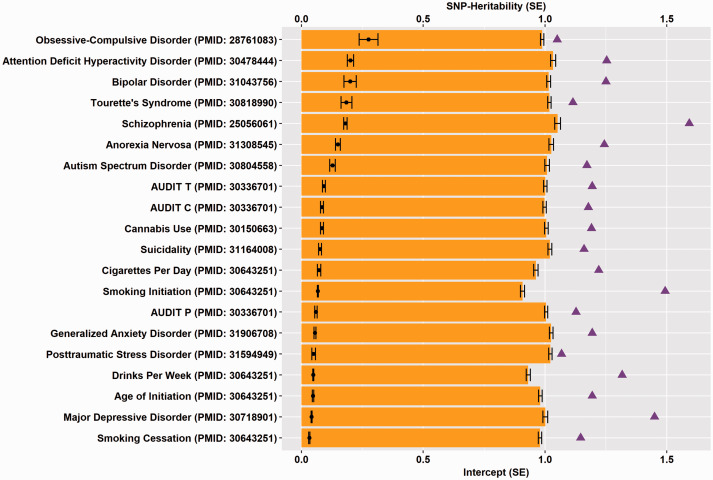
Test statistics from large-scale GWAS of psychiatric disorders (PMIDs provided) shed light on heritability and polygenicity. The orange bars represent the LDSC (linkage disequilibrium score regression) intercept, which indicates the presence of potential biases in the association analysis. The purple triangles represent the genomic inflection factor, which reflects the polygenicity of the trait when no inflation is present. The full black circles represent the SNP-Heritability. Where these statistics were not included in the associated manuscripts, they were calculated with LDSC using the 1000 Genomes Project European LD reference panel. For case-control phenotypes, SNP-*h*^2^ is plotted on the liability scale. SNP: single nucleotide polymorphism.

Large-scale GWAS have been used to understand many aspects of psychiatric disorders beyond risk locus detection. Analysis of GWAS after locus discovery is often termed “post-GWAS” analysis. Post-GWAS analyses are commonly used to follow-up risk locus discovery with additional sophisticated interpretation of GWAS signals. Some of these analyses are briefly described here. First, observed-scale heritability based on GWAS data (SNP-*h*^2^) reflects the contribution of common genetic information (rather than environmental or rare genetic factors) to the trait (Figure 1). This phenotype attribute may be conflated in case-control study designs by enrichment of cases relative to the general population prevalence. SNP-*h*^2^ also may be biased by residual population stratification from (i) higher than expected relatedness among samples or (ii) phenotype definition heterogeneity. It is understood that different functional classes of the genome disproportionately contribute to the SNP-*h*^2^. SNP-*h*^2^ estimates may then be partitioned to identify enrichment or depletion of certain functional classes of the genome such as enhancers, promoters, epigenetically regulated regions, and evolutionarily conserved regions. Another frequently-used post-GWAS method is genetic correlation whereby the per-SNP effects on one trait are regressed against the per-SNP effects of a second trait. The genetic liability for two traits may be positively, negatively, or not correlated. More sophisticated analytic tools also shed light on, or take advantage of, the polygenicity of psychiatric disorders, including (i) polygenic risk scoring (PRS, i.e. regressing weighted sum of per-SNP effects from one trait against another), (ii) Mendelian randomization (i.e. evaluating causality between phenotypes using genetic information as instrumental variables), (iii) functional annotation, fine-mapping, and co-localization to untangle polygenicity and prioritize casual risk loci, and (iv) structural equation modeling (i.e. identifying latent factors connecting phenotypes based on their genetic similarities).

## Schizophrenia

Schizophrenia (SCZ) is a severe psychiatric disorder affecting 1% of the worldwide population.^51^ Due to the high morbidity and mortality, it is also known as the “cancer of mental illness.”^52^ SCZ is diagnosed based on “positive” and “negative” symptoms. The former include hallucinations and delusions, while the latter involves avolition and withdrawal.^53,54^ Additionally, SCZ cases often present with cognitive dysfunction and deficits in executive function.^55^ Twin- and family-based studies demonstrated that individuals related to SCZ cases have an increased lifetime disease risk, ranging from 50% for monozygotic twins to 2% for first cousins.^56^ Genome-wide studies based on high-throughput technologies have revolutionized our understanding of the genetic predisposition to SCZ. In 2014, the Psychiatric Genomics Consortium (PGC) conducted a GWAS in 36,989 SCZ cases and 113,075 controls,^18^ identifying 128 independent associations spanning 108 loci and proving that SCZ architecture is highly polygenic. In an updated PGC analysis including ∼60,000 SCZ cases,^57^ more than 250 GWS risk alleles have been identified, and an SCZ PRS showed that case-control group means differ by over 2/3 of a standard deviation (0.686; *p* = 1.1 × 10^–254^). Simulations of different degrees of polygenicity across complex traits showed that SCZ could be affected at least 20,000 causal loci.^58^ Among the SCZ-associated loci, the strongest association was observed in the major histocompatibility complex region.^59^ This appears to be due to structurally diverse alleles of the complement component 4 (C4) genes that lead to a greater expression of C4A in SCZ cases.^60^ Human C4 protein is localized to neuronal synapses, dendrites, and axons, and, in animal models, C4 appears to mediate synapse elimination during postnatal development.^60^ Although these previous findings support the role of increased synapse pruning in SCZ pathogenesis, a subsequent study in human postmortem brains showed that only the smallest dendritic spines are lost in deep layer 3 primary auditory cortex of SCZ, while larger dendritic spines are retained.^61^ While mechanistic studies are essential for understanding how genetic associations contribute to disease, genome-wide studies using polygenic instruments can shed light on genetic heterogeneity in relation to phenotypic heterogeneity across SCZ cases. Comparing SCZ and bipolar disorder (BD), it was possible to identify shared risk loci, as well as loci associated that distinguish the two disorders, and to characterize polygenic composition of multiple underlying symptom dimensions.^16^ Another SCZ characteristic is the negative association with cognitive ability and educational attainment.^62^ Genome-wide analyses showed that there is a positive genetic correlation between the liabilities to SCZ and educational attainment.^63^ This apparently “paradoxical” result is not due to possible confounders (e.g. LD or assortative mating) but suggests the presence of two potential SCZ subphenotypes: one resembling high intelligence and BD, while the other is a cognitive disorder that is independent of BD.^64^

## Bipolar Disorder

BD is characterized by frequent mood swings between depressive and manic phases. The lifetime prevalence of BD is approximately 2.4% worldwide with twin-*h*^2^ of 80%.^65^ GWAS of BD have suggested a highly polygenic architecture so far comprising of 30 distinct loci associated with BD susceptibility.^66^ The most replicated risk genes are ankyrin 3 (*ANK3*) and calcium voltage-gated channel subunit alpha1 C (*CACNA1C*). Variants of these genes have been associated with white matter and total brain volume, thus implicating brain size as an intermediate phenotype. BD exists in two well-documented clinical subtypes: BD-I and BD-II.^66,67^ BD-I is distinguished from BD-II by extreme manic episodes experienced by those affected.^68,69^ The SNP-*h*^2^ of BD-I and BD-II have been estimated at 35% and 25%, respectively, with a 78% genetic overlap.^70^ This genetic overlap suggests many shared biological mechanisms contributing to each BD subtype but also suggests specific characteristics of the biological underpinnings of BD subtypes. For example, there was significantly greater relationship between (i) SCZ PRS and BD-I relative to BD-II and (ii) major depressive disorder (MDD) PRS and BD-II relative to BD-I.^66,67,70–72^ To date, the molecular mechanisms underlying these differences have yet to be robustly identified, but it is clear that studying BD as a single disorder may inflate heterogeneity and reduce power to detect the polygenic burden responsible for BD subtypes.

## Attention Deficit Hyperactivity Disorder

Attention-deficit hyperactivity disorder (ADHD) is one of the most common psychiatric disorders affecting youths in the United States.^73^ ADHD is characterized by inability to focus, impulsivity, age-inappropriate hyperactivity, and increased rates of antisocial, anxiety, mood, and substance use disorders (SUDs). The lifetime ADHD prevalence ranges from 2% to 12%^74^ with twin-*h*^2^ estimates between 74% and 80%.^75^ A GWAS of 20,183 ADHD cases and 35,191 controls identified 12 GWS risk loci for ADHD and significant differences in SNP-*h*^2^ and risk locus detection between sexes.^19,76–78^ Males are two to seven times more likely to be diagnosed with ADHD than females and largely dominate the samples included in current GWAS.^78,79^ Several hypotheses have been proposed for this sex difference. First is the scenario that female ADHD is associated with a different set of variants as compared with male ADHD.^80,81^ Another hypothesis is that females are more resilient to developing ADHD and require a higher genetic burden to present relevant diagnostic symptoms.^82,83^ Though ADHD in males and females are highly genetically and phenotypically correlated,^78,82^ GWAS are only beginning to elucidate the differences in genetic liability to ADHD across sexes. So far, there is minimal evidence that polygenic risk for ADHD contributes to, or shares underlying biology with, different co-occurring conditions or behaviors unique to each sex.^84^ To date, there have been no GWS findings for ADHD in females though this may be attributed to decreased sample size and lower population prevalence. ADHD PRS has shown positive associations with educational and cognitive outcomes,^85^ body mass index,^84^ neuroticism,^86^ externalizing behaviors (e.g. smoking, aggression, impulsivity, risk-taking),^82,84,85,87^ and interpersonal communication behaviors.^88^ In addition to sex differences, ADHD is one of the most heterogeneously diagnosed psychiatric disorders with over 116,200 diagnostic combinations according to DSM-IV and DSM-5 criterion counts. Additionally, not all criteria are required to make an ADHD diagnosis such that two individuals with ADHD may not share any diagnostic criteria resulting in a level of diagnostic heterogeneity that may confound risk locus effects in genetic studies of ADHD.^89^ For example, the commonly implicated *DAT1* underlying ADHD psychopathology has only robustly been implicated in ADHD cases without conduct-related diagnostic criteria.^75,90^

## Autism Spectrum Disorder

Autism spectrum disorder (ASD) is the term used to describe a group of pervasive neurodevelopmental disorders characterized by impairment in social and communication skills often accompanied by repetitive and restrictive behaviors.^23^ Clinical manifestation of ASD is highly heterogeneous with the majority of ASD individuals receiving a diagnosis during early childhood and adolescence.^91–95^ Heterogeneity in ASD may manifest as intellectual capabilities ranging from severe disability to high intelligence quotients^96^ or the type of social cognition impaired (i.e. person-perceptive *versus* people-perceptive social skills).^97,98^ Clinically, specific ASD diagnoses tend to be defined based on the degree of intellectual ability in the affected. Asperger’s syndrome, for example, represents some of the least severe cognitive impairments along the autism spectrum.^99,100^ Because ASD manifests as a spectrum of phenotypes, grouping individuals into ASD cases *versus* controls may introduce heterogeneity even though cases cluster together on the primary phenotype level. It has been demonstrated that reducing this spectrum phenotype heterogeneity only modestly improves genetic homogeneity in contemporary studies with large ASD sample sizes.^96^ ASD affects 1% to 1.5% of the population, and males are diagnosed more often than females.^101^ Similar to ADHD, the hypothesis of a female protective effect exists for ASD whereby females may require a greater genetic burden to develop symptoms.^101^ There is evidence that testosterone levels of males relative to females may contribute to increased vulnerability to etiological factors in ASD cases^102,103^ and putatively defines mechanistic factors inducing heterogeneity detectable by large-scale genetic studies. These sex differences have recently been implicated in investigation strategies of empathizing-systemizing and automatizing-systemizing theories of ASD heterogeneity.^97,98,104–106^ The polygenic risk for ASD has been associated with cognitive ability,^107^ various changes in DNA methylation at birth,^108^ and gray matter volume in healthy and psychiatric patients.^109^ Studies of the relationship between genetic risk for ASD and other human health and disease phenotypes have revealed interesting findings. First, ASD genetic risk indeed predicts ASD severity; however, ASD PRS do not cleanly stratify individuals into more clinically severe ASD symptom criteria.^107,110^ This observation suggests that though phenotypic ASD subtypes exist, they may not appropriately stratify ASD for genetic studies.^96^ Second, the genetic risk for ASD in ASD unaffected individuals (i.e. unaffected individuals carrying ASD) associates with features of healthy neurodevelopment.^98,106^

## Posttraumatic Stress Disorder

Posttraumatic stress disorder (PTSD) affects individuals who have experienced, witnessed, or been confronted with an event involving actual or threatened danger. This required environmental component of PTSD makes it unique among DSM-5 disorders without such required etiologies. Diversity among traumatic experience adds substantial heterogeneity to PTSD cases, and this diversity can be detected with GWAS.^27,30^ Given the abundance of PTSD in veteran populations, exposure to combat-related experiences has garnered much attention. Two large studies of PTSD estimated SNP-*h*^2^ of 2% to 5% in the PGC international meta-analysis and 6.4% to 10.1% in the Million Veteran Program (MVP).^30^ These cohorts represent different trauma exposures with MVP comprised mostly of males exposed to military combat and PGC comprised of international sex-balanced PTSD cases exposed primarily to civilian traumas. Traumatic events vary by sex, demography, and socioeconomic status. Males have higher rates of overall trauma exposure, yet females are more likely to develop PTSD following similar trauma, resulting in approximately doubled lifetime PTSD prevalence in U.S. females (8%) relative to males (4.1%).^111,112^ Furthermore, sexual trauma is more prevalent among females, and males are more likely to experience nonsexual assaults, death/injury, and military combat. Specific traumas also appear to convey different magnitudes of risk for PTSD.^113^ Sex differences are reflected in sex-stratified GWAS where the SNP-*h*^2^ of PTSD in males was no different from zero regardless of ancestry and the SNP-*h*^2^ of PTSD in females was 8% to 18% with African ancestry individuals demonstrating the highest SNP-*h*^2^ estimates.^27,114^ Additional heterogeneity of PTSD can be seen among the responses to trauma evaluated for PTSD diagnosis.^22,30^ Including the PTSD symptom criteria of reexperiencing, avoidance, negative emotional symptoms, and hyperarousal, there are 636,120 possible combinations by which a person may be diagnosed with PTSD.^115^ Several loci (localizing near genes *MAD1L1*, *TCF4*, and *TSNARE1*) have been implicated across symptoms, lending support for these loci as putative targets for PTSD treatment. Lastly, there is considerable heterogeneity in the longitudinal course of PTSD, with distinct trajectories of symptom onset, severity, and remission that may be in part related to trauma context, sex, access to care, and other unmeasured influences.^116,117^ Future work in PTSD genetics will surely begin addressing how differences in genetic risk also influence PTSD trajectory and prognosis.

## Major Depressive Disorder

MDD is the unceasing depressive or low mood lasting for more than two weeks accompanied by disturbances in weight, circadian rhythms, elevated negative emotions, and self-debilitating thoughts. The lifetime prevalence of MDD in the United States is approximately 20.6%,^118^ and twin-*h*^2^ is estimated between 30% and 40%.^119^ MDD is associated with socioeconomic burden and all-cause mortality and is thus a leading cause of disease burden worldwide.^120^ Phenotypic heterogeneity of MDD is primarily driven by sex differences; the lifetime prevalence of MDD in females is 26.1% and in males is 14.7%.^118^ To date, GWAS have identified many loci conferring risk for MDD, the largest of which (*N *=* *807,553 individuals) detected 102 MDD risk loci.^24,32,121–123^ Though effective sample sizes continue to increase, SNP-*h*^2^ estimates converge between 8.7% and 8.9%.^24^ Heterogeneity in MDD may be evident by the presence of five DSM-IV diagnostic criteria and lower SNP-*h*^2^ estimates in males *versus* females.^124^ Furthermore, many of the single-item criteria for an MDD diagnosis (e.g. “nerves, anxiety, tension or depression,” and “self-reported depressive symptoms with associated impairment” items in the UK biobank) overlap with multi-item diagnostic instruments for other disorders such as BD and SCZ.^24,121,125^ These shared diagnostic criteria may reduce accuracy of PRS in stratifying MDD individuals and introducing heterogeneous genetic associations. It is important to note that MDD, like other psychiatric disorders, exists as a spectrum rather than clear case-control distinction, and the reduction of MDD into such a binary classification system may introduce heterogeneity among case and control categories.

## Obsessive-Compulsive Disorder

Obsessive-compulsive disorder (OCD) is characterized by recurrent, intrusive, or unwanted thoughts, images, or impulses that provoke anxiety and actions to ameliorate that anxiety. OCD cases display concerns about contamination, responsibility for harm or injury, unacceptable thoughts that are often sexual and/or religious in nature, and symmetry, completeness, and the need for things to be “just right.” Compulsive behaviors to neutralize anxiety include excessive cleaning/hygiene, repeated checking, or other ritualized thoughts and behaviors. The lifetime prevalence of OCD is 1% to 3%,^126,127^ and twin-*h*^2^ is estimated to be 48%.^128^ To date, OCD GWAS have not detected any GWS risk loci, but genetic data estimate SNP-*h*^2^ at 28%.^129^ There are notable sex differences observed in clinical presentations of OCD, with males comprising roughly two thirds of childhood-onset cases and reporting a higher incidence of obsessions related to religious/sexual thoughts and symmetry themes.^127,130^ Females are more likely to present with late-onset OCD and report higher rates of precipitating events (e.g. pregnancy and childbirth), exacerbation of symptoms with hormonal events, and higher rates of comorbid eating disorder.^127,130^ Obsessive themes among females with OCD tend to center around hygiene.^127^ Large-scale GWAS of OCD in sex-stratified cohorts failed to detect significant SNP-*h*^2^ in males but estimate significant SNP-*h*^2^ in females (30%). Sex-stratified OCD GWAS are underpowered to formally test the genetic correlation between them; however, per-SNP effect sizes tend to positively associate. One study suggested that SNP-*h*^2^ may vary across different age groups with greater SNP-*h*^2^ estimates in OCD of younger individuals (*h*^2^=0.43) versus older individuals (*h*^2^ not significant).^131^ OCD has received considerable attention due to its relationship with other psychiatric disorders. In a familial co-aggregation study, first-degree relatives of OCD patients had more than double the risk for BD (relative risk (RR) confidence interval (CI) = 2.68–3.04), MDD (RR CI = 2.58–2.67), ASD (RR CI = 2.10–2.71), ADHD (RR CI = 2.07–2.32), and SCZ (RR CI = 1.86–2.09).^132^ Exploring how OCD relates to these other psychiatric disorder revealed overlapping exomes and polygenic risk between OCD and SCZ and identified *DMN3* as one suggestive link between the two disorders.^133^

## Tourette’s Syndrome and Chronic Tic Disorder

Tourette’s syndrome (TS) is typically diagnosed before 18 years of age and requires two or more motor and at least one phonic tic lasting more than one year.^134^ The related diagnosis, chronic tic disorder (CTD), requires the presence of two or more of either motor or phonic tics, but not both. TS has a prevalence of 0.3% to 0.8% and occurs more frequently in males, at a ratio of approximately 3.5:1,^135^ with similar prevalence reported for CTD.^136,137^ Though males tend to be diagnosed more often than females, females experience greater day-to-day burden of more severe tics.^138–140^ The largest GWAS of TS (*N *=* *14,307) identified a single GWS variant and 39 suggestive associations.^33^ Estimates of TS SNP-*h*^2^ range from 21% to 58%,^33,131^ with twin-*h*^2^ estimates up to 60%.^131^ TS PRS were predictive of clinical status in independent samples, with probands from multiplex families showing higher loading than those from simplex families.^33,141^ Individuals with CTD have elevated TS PRS relative to controls. Childhood neurodevelopmental disorders (e.g. ADHD, ASD, OCD, and TS/CTD) share elevated rates of comorbidity as well as shared subphenotypes (e.g. executive functioning, impulse control, intrusive thoughts, repetitive behaviors, and rigid adherence to routines), which pose challenges for both clinical subphenotyping and for understanding genetic effects. TS PRS have been associated with (i) the presence, but not chronicity of tics and (ii) the severity of symptoms associated with comorbid conditions.^142^ Much of the heterogeneity associated with TS and CTD stems from the relationship between TS/CTD and OCD and ADHD. Two TS subphenotypes have been detected, the symmetry subphenotype and disinhibition subphenotype, which have distinct genetic architectures not fully understood due to strict adherence to DSM-based diagnoses.^143^ The symmetry subphenotype was positively predicted by TS (but not OCD or ADHD) PRS, while the disinhibition subphenotype was predicted by OCD (but no other) PRS. In a cross-trait gene-based study of OCD and TS, *CADM2*, *LY6G6F*, *MEGT1*, and *APOM* were identified as GWS loci but were not detected in other pairwise neurodevelopmental disorder gene-based analyses^142^ To disentangle and specify genetic relationships among these disorders, future studies may benefit from (i) the incorporation of shared intermediate phenotypes (ii) *post hoc* conditioning of analyses to remove shared or nonspecific effects, and (iii) investigating molecular mechanism involving identified gene targets shared and differentially expressed between TS and other psychiatric disorders (e.g. OCD).^143^

## Anxiety Disorders

Anxiety disorders in DSM-5 include separation anxiety, selective mutism, specific phobias, panic disorder, agoraphobia, and generalized anxiety disorder (GAD). The lifetime prevalence of anxiety is estimated at 31%.^144,145^ Twin studies suggest anxiety disorder twin-*h*^2^ of 42%.^146^ Anxiety disorders are quite heterogeneous, and many subtypes are ubiquitously more prevalent in females.^147^ GAD is perhaps the most thoroughly investigated by large-scale GWAS. GAD is defined as the presence of excessive anxiety and worry about a variety of topics, events, or activities, typically lasting more than six months. The excessive worry associated with GAD is often to the point where the affected cannot control themselves. Physical symptomology often varies between cases and may include edginess, impaired concentration, empty mindedness, irritability, muscle aches, and sleeplessness.^148–150^ Recent GWAS of GAD in >200,000 U.S. military veterans identified risk loci shared between GAD and SCZ and BD.^25^ Collectively, the polygenic architecture of GAD-2 (a two-item GAD symptom criterion checklist) significantly overlapped with MDD, neuroticism, and PTSD.^25^ Though significant overlap exists between various measures of anxiety disorders (anxiety case-control,^151^ GAD-2,^25^ and GAD-7^152^), these overlaps are not perfect, suggesting that the distribution of anxiety symptoms in study cohorts may be readily detected in the associated genetic data. Anxiety disorders demonstrate substantial heterogeneity based on age of the ascertained cohort.^153,154^ Two anxiety disorders, separation anxiety and selective mutism, were once thought to be exclusively childhood disorders, but it is now accepted that children and adults may receive these diagnoses. There also is evidence that environmental heterogeneity, such as childhood maltreatment, moderate polygenic risk in genetic studies of anxiety disorders.^155–157^

## Suicidality

Worldwide, more than 1 million people complete suicide every year making it the 10th leading cause of death in the United States (12.6 deaths per 100,000).^158^ Nonfatal suicidal behaviors also are a consistent emotional and economic burden. Suicidal ideation, plans, gestures, attempts, and completed suicides represent a continuum of suicidal behavior. There is one death by suicide for every 25 attempts,^159^ and some of attempts are severe enough to require medical attention and may have long-lasting sequelae. Having a psychiatric disorder is a major risk factor for suicidal behaviors and individuals affected by a mental illness may represent at least 90% of the people who have died by suicide.^160^ However, most people with mental disorders do not die by suicide, and the risk of suicide is 5% to 8% for several mental disorders.^161^ According to the stress-diathesis model, the risk for suicidal acts is determined a stressor (such as psychiatric illness) and a diathesis, such as a tendency to experience more suicidal ideation and be more likely to act on suicidal feelings.^162^ Twin, family, and adoption studies identified a 30% to 50% *h*^2^ which appears to be partially independent from psychiatric disorders.^163^ GWAS have just started to investigate suicidal behaviors in relatively large cohorts.^67,164–167^ These analyses were conducted on cohorts with different characteristics including general population, military personnel, and individuals affected by psychiatric disorders. Although few risk loci were identified, a consistent genetic overlap has been observed between MDD and suicidal behaviors.^67,164–167^ A recent multivariate genome-wide interaction study detected that genetic risk for suicidal behaviors is partially moderated by multivariate gene interactions linking comorbid substance dependences with suicidal ideation.^168^ This suggests that the phenotypic heterogeneity among individuals experiencing suicidal behaviors increases the genetic complexity of these traits. Genome-wide approaches have the potential to disentangle the diverse characteristics observed among individuals experiencing suicidal feelings and committing a suicidal attempt. However, much more informative cohorts are needed to achieve a comprehensive understanding of the molecular basis of suicidal behaviors.

## Feeding and Eating Disorders

Eating disorders are classified as abnormal eating episodes occurring intermittently or frequently and lasting for more than three months. DSM-IV recognized three primary diagnoses: anorexia nervosa (AN), bulimia nervosa (BN), and eating disorders not otherwise specified (called EDNOS; diagnosed by omission of symptom criteria for AN and BN). The prevalence rates of eating disorders are affected by socioeconomic status. Females experience higher prevalence than men, non-Hispanic populations of European descent tend to have higher prevalence than individuals of other ethnicities, and there is some evidence that family income may contribute to eating disorder prevalence.^169^ Psychiatric comorbidities are common among eating disorder cases, including MDD, anxiety, and OCD.^170^ The twin-*h*^2^ of eating disorders varies from 40% to 60%.^171^ The SNP-*h*^2^ of eating disorders (cases included AN and BN; *N*∼14,000) was 20% in early studies of this class of disorders.^172^

### Anorexia Nervosa

AN has a prevalence of 0.8% and is characterized by restricted eating, weight loss, difficulties maintaining an age- or height-appropriate body weight, and distorted body image. Genetic studies have mostly focused on AN likely due to the higher population prevalence compared to other eating disorders. The most recent and largest GWAS of 76,644 individuals detected eight risk loci using diagnostic criteria from DSM-III to DSM-5. The study reported SNP-*h*^2^ of approximately 11% and relatively high genomic lambda (1.22) but appropriate LD intercept which collectively provide evidence of high polygenicity. Partitioning the SNP-*h*^2^ for mouse model cell types identified spiny and pyramidal neurons of the hippocampus, which are responsible for feeding behavior and impetus. Additionally, reported genetic correlation of AN with psychiatric disorders and metabolic dysregulation parallels epidemiologically observed comorbidities.^31^

### Bulimia Nervosa

BN is characterized by cycles of bingeing and caloric compensatory behaviors, such a vomiting, and its prevalence is 0.28%.^169^ While GWAS have provided much needed resolution of genetic liability to AN, the genetic liabilities of BN, and other eating disorders are less clear. Larger sample sizes will indeed be required to detect genetic correlations, SNP-*h*^2^, and underlying biology associated with BN such that therapeutic and diagnostic interventions may be developed.^171^

## Substance Use Disorders

SUDs are characterized by uncontrolled desire for excessive substance intake and inability to reduce the frequency of consumption. According to the WHO, there are more than 180-million drug users worldwide.^173^ DSM-5 has expanded SUD definition to include gambling disorders and combines the concepts of substance abuse and dependence, though there is evidence of shared and specific genetic effects for these traits.^174^ Commonly studied substances include alcohol, stimulants (e.g. amphetamines and cocaine), tobacco, and opioids.^175^ Phenotypic heterogeneity in SUDs stems from various drug seeking patterns, environmental factors, pharmacokinetic and pharmacodynamic processes, and psychiatric comorbidities.^173^ The characterization of behavioral and psychiatric traits related to the use and abuse of addictive substances in large cohorts remain challenging due to several factors, such as the hypothesized differences in recreational versus prescription use and abuse and the societal stigma associated with use of certain substances over others. These barriers are being overcome through large consortium and biobank efforts, but studies of some substances (e.g. opioids and cocaine) remain underpowered to make robust conclusions about underlying biology.

### Alcohol

Individuals with alcohol use disorder (AUD) had high comorbidity with other psychiatric disorders, while alcohol consumption has a much lower genetic correlation with psychiatric disorders.^176^ AUD is measured by dependence on extreme alcohol consumption and has a twin-*h*^2^ of 50%.^177^ SNP-*h*^2^ for alcohol dependence averages around 10%. GWAS for alcohol dependence using DSM and alcohol consumption with the AUD Identification Test converge on variants in the alcohol metabolizing gene *ADH1B* and other genes (*GCKR*, *SLC39A8*, *FTO*, *ADH4*, *SIX3*, and *DRD2*) with shared biological functionality. Only half of the genes overlap between alcohol consumption and AUD suggesting distinct etiologies between consuming/using alcohol and being dependent on its effects.^178^

### Nicotine

Smoking cigarettes, whose primary substance is nicotine, is a complex phenotype ranging from initiation, consistent pattern, dependence, termination, and reversion. The family-*h*^2^ for nicotine dependence is measured to be 75%.^179^ GWAS have consistently replicated a region on chromosome 15 consisting of *CHRNA3*, *CHRNA4*, and *CHRNA5* which explain 4% to 5% of the variance in smoking-related phenotypes. These results are analogous to other SUDs, drawing attention to biological heterogeneity varying with the severity of dependence.^174,180,181^

### Opioids

Like AUDs, assessing differences between opioid use and dependence proves essential to understanding the polygenic architectures of these traits.^20^ A recent study comparing opioid-exposed versus unexposed controls detected SNP-*h*^2^ of 28%.^174^ A study conducted in 10,544 OUD cases and 72,163 opioid-exposed controls from the MVP cohort identified *OPRM1* Asn40Asp (rs1799971) as a significant risk locus, also showing genetic correlation with multiple substance use traits and psychiatric illnesses and possible causal effects on OUD risk from tobacco smoking, major depression, neuroticism, and cognitive performance.^34^

### Cannabis

The family-wise *h*^2^ of cannabis use is 45%, and recent GWAS of 184,765 individuals reported SNP-*h*^2^ of 11%. Genetic risk for cannabis use was positively genetically correlated with MDD and SCZ, risk-taking behavior, and neuroticism.^182^ Age at cannabis initiation also appears to be moderately heritable, and the significant association with *ATP2C2* is consistent with the role of calcium signaling mechanisms in the propensity to cannabis use.^183^ In a GWAS of cannabis dependence, there is a consistent overlap with potential genetic factors contributing to major depression and SCZ.^184^

## Conclusions and Future Directions

GWAS have contributed major advances to our understanding of the polygenic architecture of psychiatric disorders. However, the phenotypic and genetic heterogeneity described herein contribute to complicate the translation of genetic data into clinical practice. For example, sex differences are ubiquitous across psychiatric disorders but are only recently being investigated with genome-wide methods. Unfortunately, stratifying by sex drastically reduces sample size for a GWAS, but large-scale genomics consortia are rapidly collecting suitable sample sizes to make these analyses more feasible and reliable. Until then, the community may consider focusing attention on several additional sex-specific topic areas including X-chromosome studies and regulatory/expression studies of risk loci. X-chromosome association studies are still relatively novel and require additional consideration of dosage differences between sexes but hold great potential for uncovering differential disorder risks in males and females.^185–187^ Furthermore, regulatory mechanisms have been identified as likely contributors to the sex differences in many disorders, but these processes remain vastly underinvestigated.^188–190^ These studies will be particularly informative for understanding how genes discovered by GWAS are expressed in each sex.

We have summarized how phenotypic heterogeneity greatly influences genetic heterogeneity in psychiatry. The RDoC paradigm may help considerably reduce this heterogeneity by focusing on biologically tractable processes, measurements, and/or mechanisms rather than diagnoses dependent on multi-item symptom checklists.^49^ It is important to note that some of the existing GWAS of psychiatric disorders likely already incorporate some aspects of RDoC (e.g. (a) hyperarousal and reexperiencing symptoms of PTSD diagnosis^22^ and (b) studying hallucinations as a representative symptom of psychotic disorders^191^).

GWAS of psychiatric disorders have elucidated thousands of risk loci contributing to disease etiology and generated countless testable hypothesis addressing psychiatric disorder heterogeneity, comorbidities, and cross-species interactions (e.g. microbiome-brain interactions). GWAS data, and therefore the resulting post-GWAS analyses, may be influenced by phenotype and sample heterogeneity which both have document effects on detection of the polygenic architecture of a trait. It is well understood that the polygenicity of a disorder in one population may not reflect the polygenicity of the same disorder in an external population. This observation means that findings from well-studied European populations may not, and indeed do not,^192^ translate effectively to non-Europeans and supports a discipline-wide effort to close this gap by studying psychiatric disorder polygenic architectures in other populations.
